# A genes and health recall study of intrahepatic cholestasis of pregnancy and cholestatic liver disease

**DOI:** 10.1038/s43856-025-01228-4

**Published:** 2025-12-23

**Authors:** Maria Constantinides, Joseph Gafton, Ana Cristina Angel Garcia, Genes & Health Research team, Eamonn Maher, Shabana Chaudhary, Joseph Gafton, Karen A. Hunt, Shapna Hussain, Kamrul Islam, Mohammed Bodrul Mazid, Elizabeth Owor, Jessry Russell, Nishat Safa, John Solly, Marie Spreckley, David A. Van Heel, Jan Whalley, Ishevanhu Zengeya, Emily Mantle, Shaheen Akhtar, Samina Ashraf, Dan Mason, John Wright, Daniel MacArthur, Michael Simpson, Richard C. Trembath, Gerome Breen, Raymond Chung, Sang Hyuck Lee, Omar Asgar, Joanne Harvey, Karen Tricker, Caroline Winckley, Hanifa Khatun, Amna Asif, Claudia Langenberg, Grainne Colligan, Ceri Durham, Bill Newman, Ahsan Khan, Hilary Martin, Teng Heng, Matt Hurles, Vivek Iyer, Georgios Kalantzis, Vladimir Ovchinnikov, Iaroslav Popov, Klaudia Walter, Panos Deloukas, David Collier, Ana Angel, Saeed Bidi, Fabiola Eto, Chris Griffiths, Sam Hodgson, Benjamin M. Jacobs, Rohini Mathur, Caroline Morton, Asma Qureshi, Stuart Rison, Annum Salman, Miriam Samuel, Moneeza K. Siddiqui, Daniel Stow, Sabina Yasmin, Julia Zöllner, Sheik Dowlut, Sarah Finer, Peter H. Dixon, Catherine Williamson, Kenneth Linton, Sarah Finer, Upkar S. Gill, Julia Zöllner

**Affiliations:** 1 Wolfson Institute of Population Health, Queen Mary University of London, London, UK; 2 Imperial College London, London, UK; 3 Centre for Cell Biology and Cutaneous Research, Blizard Institute, Barts and the London School of Medicine and Dentistry, Queen Mary University of London, London, UK; 4 Centre for Immunobiology, Blizard Institute, Barts and The London, School of Medicine & Dentistry, Queen Mary University of London, London, UK; 5 University College London, London, UK; 6 Aston University, Birmingham, UK; 7 Blizard Institute, Queen Mary University of London, London, UK; 8 Bradford Teaching Hospitals NHS Foundation Trust, Bradford, UK; 9 Garvan Institute, Darlinghurst, NSW Australia; 10 King’s College London, London, UK; 11 Manchester University Hospitals, Manchester, UK; 12 Precision Healthcare University Research Institute, Queen Mary University of London, London, UK; 13 Social Action for Health (charity), London, UK; 14 University of Manchester, Manchester, UK; 15 Waltham Forest Council, London, UK; 16 Wellcome Sanger Institute, Hinxton, UK; 17 William Harvey Research Institute, Queen Mary University of London, London, UK

**Keywords:** Genetics, Genetic testing

## Abstract

**Background:**

Cholestatic liver disease disproportionately affects South Asians, yet they remain underrepresented in genomic studies. This recall study aimed to recall volunteers from a British South Asian genetic cohort that were considered to be at high risk of cholestatic liver disease based on their genotype or phenotype.

**Methods:**

Cases were defined as participants with rare (minor allele frequency <1%) heterozygous loss of function (LoF) variants in *ABCB4* and *ABCB11* (genotype re-call) or with a previous intrahepatic cholestasis of pregnancy (ICP) diagnosis (ICD10 O26.6). Cases were matched 1:1 to controls. A detailed medical and family history was taken along with fasting anthropometric and transient elastography (TE) measurements and blood samples.

**Results:**

Out of 22 eligible volunteers, 9 (41%) participate in the recall (8/9 genotype and 1/9 phenotype recall). Among the recalled cases there are 5 *ABCB4* LoF, 3 *ABCB11* LoF, and 1 ICP phenotype. Of these, 5/9 (55.6%) exhibit findings suggestive of liver involvement (genotype re-call). Specifically, 2/5 (50%) have increased liver stiffness on TE with one also demonstrating abnormal liver blood tests. 2/5(40%) report at least 2 cholestatic symptoms and an additional 1/5 (20%) demonstrates abnormal liver blood tests without increased liver stiffness.

**Conclusions:**

This study shows findings suggestive of liver involvement in 55.6% of volunteers, underscoring the potential of rare heterozygous *ABCB4/11* variants as markers for identifying individuals at high risk of developing cholestatic liver disease. Consequently, individuals at higher genetic risk benefit from monitoring, personalised treatment and prevention strategies for cholestatic liver disease.

## Plain language summary

We aimed to identify British South Asians at high risk of liver disease due to specific genetic factors, such as issues with bile production or liver problems during pregnancy. We invited these individuals to a clinic, where we collected their medical and family history, conducted liver blood tests, and performed a scan to check for early signs of liver scarring. We found that nearly two out of three participants had undetected liver disease. This finding suggests that genetic factors are linked to developing liver disease, highlighting the importance of early detection and monitoring for those at risk.

## Abstract (web summary)

Constantinides et al., perform a recall study from a British South Asian genetic cohort to explore the genetic and phenotypic risk of cholestatic liver disease. 55.6% of participants with rare heterozygous ABCB4/ABCB11 variants or a history of intrahepatic cholestasis of pregnancy showed evidence of liver involvement, highlighting the utility of genetic screening and monitoring.

## Introduction

Cholestatic liver disease is a growing cause of morbidity and mortality worldwide as it can lead to liver fibrosis and liver cirrhosis^1–3^. In the United Kingdom, there has been a 400% increase in mortality due to liver disease over the last 50 years with an acceleration in liver disease rates compared with other major diseases^4^. In South Asian populations liver diseases are more common, although they often remain undiagnosed and under-investigated^5, 6^. Cholestatic liver disease encompasses a broad range of diseases characterised by jaundice and cholestasis which can result in end-stage liver disease, cirrhosis, and other-severe liver-related complications. Symptoms of cholestatic liver disease include pruritus, abdominal pain (epigastric or right upper quadrant pain (RUQ)), steatorrhea, jaundice, dark urine and pale stools^1^. Cholestasis biochemically is characterised by an increase in alkaline phosphatase (ALP) or gamma‑glutamyl transferase (GGT) an increase in bilirubin, which can occur at a later stage due to decrease in bile flow and, when measured, elevated serum bile acid concentrations^7^.

Genes involved in bile formation, such as *ABCB4* (encodes canalicular phosphatidylcholine floppase) and *ABCB11 (*encodes bile salt export pump (BSEP)) have been associated with cholestatic liver disease ^8–10^. Homozygous variants in *ABCB4* and *ABCB11* are associated with a variety of phenotypes ranging from mild cholestasis to severe familial cholestasis, such as progressive familial intrahepatic cholestasis (PFIC)^11^. Specifically, *ABCB4* variants have been shown to predispose to adult biliary cirrhosis, gallstone, gallbladder and bile duct carcinoma, drug induced cholestasis and low phospholipid associated cholelithiasis (LPAC) ^10–14^. It appears that carriers of heterozygous variants in genes associated with disease can develop cholestatic disease with a spectrum in the disease phenotype and onset^11, 15^. For example, in genetically susceptible individuals the altered physiological state of pregnancy, that is accompanied by an increase in serum concentrations of oestrogen and progesterone and their metabolites, can reveal a cholestatic phenotype (i.e., intrahepatic cholestasis of pregnancy (ICP), the commonest gestational liver disease)^16^. The cause of ICP is multifactorial but a study showed that 12.8% of mothers, 15.9% of sisters and 10.3% of daughters were affected, highlighting a strong genetic link^17^. Furthermore, a European ancestry study demonstrated that 20% of severe ICP cases harboured a pathogenic/likely pathogenic heterozygous variant in *ABCB4* or *ABCB11*(18). Not only is ICP associated with adverse pregnancy outcomes^11^, women with ICP also have an increased risk of developing hepatobiliary diseases such liver cirrhosis, hepatitis C, cholangitis and gallstones^18–23^. In actual fact, the presence of *ABCB4* variants in ICP patients increased the risk of developing hepatobiliary disease in an Icelandic population^24^. Similarly, our recent work identified novel variants associated with cholestatic liver disease and demonstrated an increased heterozygous rare variant burden associated with ICP in a British Bangladeshi and Pakistani population^11^. For the treatment of cholestasis, ursodeoxycholic acid (UDCA) is the most commonly used drug with other medications, such as rifampicin, cholestyramine and fibrates being used^25^. Medical management with UDCA is considered to be the first line therapy in all cases with PFIC, with some cases with PFIC benefiting from surgical biliary diversion or ileal bile acid inhibitor treatment^26, 27^, with liver transplantation being used when treatment fails ^14, 25, 28^.

Genes & Health is a community-based genetic study of health and disease in British Bangladeshi and Pakistani people, who are underrepresented in existing cohorts. This study recalls existing volunteers already recruited to Genes & Health and in whom linkage to health and genetic data is already established. The individuals recalled were previously identified by our group^11^ to be at high risk of cholestatic liver disease based on their genotype (rare heterozygous loss of function (LoF) variants in *ABCB4* or *ABCB11*) or phenotype (ICP).

Our aim was to identify cases at highest genetic risk of cholestatic liver disease and correlate genetic risk with clinical findings. We hypothesised that heterozygous LoF variants or a previous history of ICP would predispose to cholestatic liver disease and may be detectable in advance of symptomatic disease onset. Our findings in this study suggest liver involvement in 55.6% of these cases emphasising the role of rare heterozygous *ABCB4/11* variants as potential markers for identifying those at elevated risk of cholestatic liver disease.

## Methods

### Genes & health study

Genes & Health volunteers are currently recruited in three sites across the United Kingdom (East London, Bradford and Manchester), in community (mosques, markets and libraries) and health care (NHS GP practices, outpatient clinics) settings. DNA (saliva) is taken for SNP (single nucleotide polymorphism) array genotyping and exome sequencing^29^. Genes & Health combines electronic health record data, with primary and secondary care records and genetic data as previously described^29^.

### Study design

A recall-by-genotype and a recall-by-phenotype approach was implemented in Genes & Health participants with exome sequencing data available. The study was conducted between April 2023 – August 2023. At the time of the study, exome sequencing data was available for 5236 volunteers reporting parental relatedness. Participants were recruited and seen in East London Genes & Health. Participants received compensation for their time provided as per Genes and Health guidelines.

### Participant recruitment

**Fig. 1:**
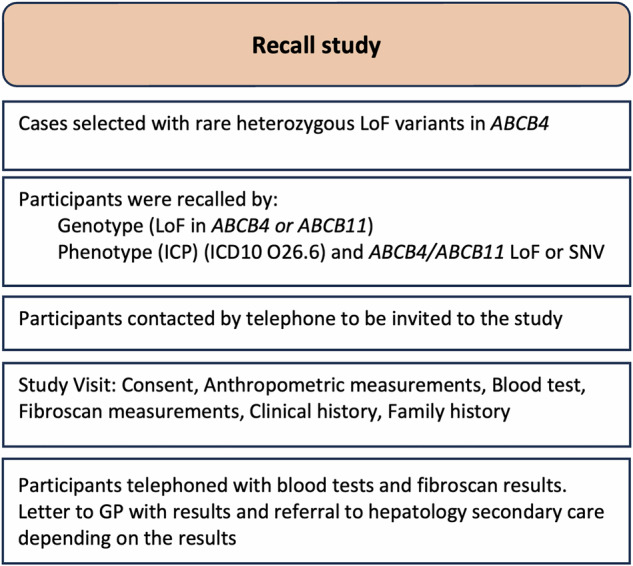
Re-call study participant selection. Abbreviations: GP general practitioner, ICD10 International classification of diseases 10th revision, ICP intrahepatic cholestasis of pregnancy, LoF loss of function, SNV single nucleotide variant.

Figure 1 describes targeted recruitment to this recall study. Cases with rare (minor allele frequency (MAF) < 1%) heterozygous LoF variants in genes highly associated with disease - *ABCB4* and *ABCB11* (see supplementary Table 1) were recalled. Importantly, none of these cases carried ATPB1 variants that could have contributed to disease. LoF variants impair the functionality of human protein-encoding genes. Homozygous LoF variants are associated with severe cholestatic liver disease phenotypes, therefore heterozygous mutations with ensuing haploinsufficiency are likely to increase susceptibility to disease and were considered high risk for the development of a sub-clinical cholestatic liver disease phenotype. Additional cases were selected as those with ICP phenotype (coded through ICD10 O26.6 in electronic health records) with either rare heterozygous single nucleotide variants (SNV) or LoF variants (see supplementary Table 2).

Cases were matched with a 1:1 ratio to healthy controls. Controls were Genes & Health volunteers matched according to age (+/− 5 years), sex, and ethnicity (British Pakistani or British Bangladeshi). Exclusion criteria for controls were anyone with pathogenic variant in *ABCB4/ABCB11/ATP8B1* (genes associated with cholestatic liver disease), known cholestatic liver disease, liver cirrhosis or carcinoma, bile duct carcinoma, hepatitis B/hepatitis C, gallstones, covid infection in the preceding 6 weeks, any new medication introduced in the last 3 months causing cholestatic side effects, such as jaundice, dark urine, pale stools, pruritus.

### Study Visit Appointment

Participants fasted for a minimum of 8 h. A detailed medical history and family history (see supplementary Table 3) was taken by a clinician (MC or JG). Current symptoms referred to symptoms experienced by the participants within one month from recall appointment. At the visit anthropometric measurements (height, weight, waist circumference and hip circumference) and bioimpedance were taken. Blood analysis included plasma aliquots and blood cell RNA preservation (Paxgene). Quantitative analysis included full blood count, urea and electrolytes, bone profile, liver blood tests (alanine transaminase (ALT), aspartate transaminase (AST), GGT, enhanced liver fibrosis (ELF) test^30^, bile acid concentration, virology screen (Hepatitis B surface Ag (HBsAg), Hepatitis C IgG, HIV 1/2 antibodies), lipids, fasting glucose and glycated haemoglobin (Hba1c).

As per recommendations of EASL guidelines, to assess hepatic fibrosis non-invasive tests (NITs), such as Fibrosis 4 index (FIB-4) score^31^, enhanced liver fibrosis (ELF) test^30^, and transient elastography (TE) were performed. A FIB-4 score of <1.3, an ELF score <9.8 were used to rule out hepatic fibrosis as per EASL guidelines^32^.

To assess hepatic fibrosis and hepatic steatosis, TE using FibroScan compact 530 (Echosens) was performed. Values of liver stiffness measurement (LSM, in kPa)((surrogate marker of liver fibrosis)^33^ and liver CAP (in dB/m) (surrogate marker of liver steatosis)^34^ were obtained. When measuring LSM to ensure reliable results IQR/Median (%) shall remain ≤ 30%^35^. No quality criteria have been defined when measuring CAP. As per EASL guidelines^32^, LSM < 8 kPa was used to rule out fibrosis, LSM 10 – 12 kPa indicating probable advanced chronic liver disease and LSM > 12–15 kPa to rule in compensated advanced chronic liver (cACLD). Cut off points differ with aetiology but provide an overall approximation^32^. Although there has not been a general consensus for cut-off values, CAP values above 275 dB/m were used to indicate steatosis as per EASL guidelines^32^.

### Statistics and Reproducibility

Statistical analysis was performed using SPSS for Mac Version 28 and 29 and statistical significance was set at *p* < 0.05. Normality of data was assessed using histograms and the Shapiro Wilk test. Continuous variables following normal distribution were analysed using independent t-test. Continuous variables not following normal distribution were analysed using the Mann-Whitney Test. Nominal data were analysed using Fischer’s exact test. Replicate samples were not collected, as all analyses were conducted in a United Kingdom Accreditation Service (UKAS)-accredited laboratory.

### Ethical approval

Genes & Health ethical approval was granted by the South East London National Research Ethics Committee (14/LO/1240) in 2014. This recall study operated with ethical approval from the West of Scotland Research Ethics Service (22/WS/0109).

### Reporting summary

Further information on research design is available in the Nature Portfolio Reporting Summary linked to this article.

## Results

**Fig. 2:**
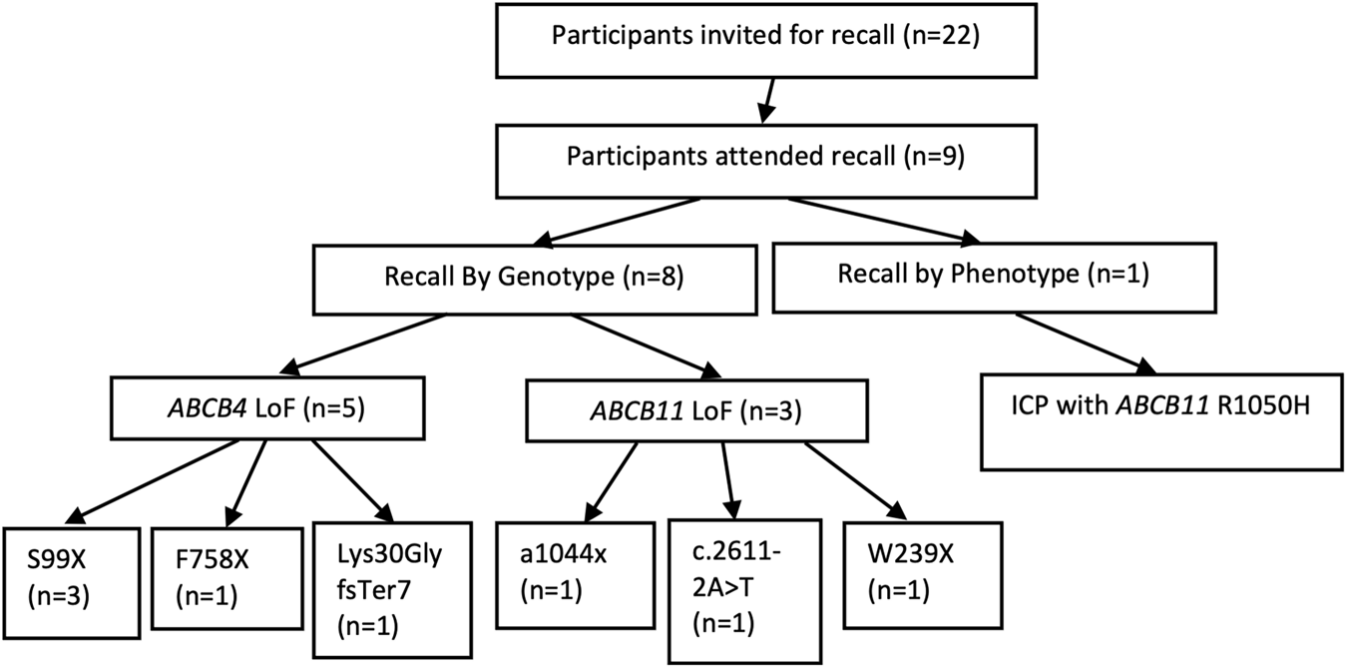
Schematic flow chart of participants that attended recall. Abbreviations: ICP intrahepatic cholestasis of pregnancy, n=number of volunteers, LoF loss of function.

Out of 22 eligible cases, nine (41%) attended a clinic appointment (six females, and three male cases) that were matched to nine controls (Table 1). Out of the 13 cases who were invited to participate but did not attend, seven (54%) showed no interest in participating, three (23%) agreed to participate but did not attend their appointments, two moved (15%) outside London and one (8%) was interested to participate but was abroad during the study period. Among the cases there were five *ABCB4* LoF, three *ABCB11* LoF, and one ICP phenotype. Figure 2 illustrates the cases who attended the clinic for recall and their variants. The clinical and demographic characteristics of cases and controls are shown in Table 2. No statistically significant difference was found in the demographics between cases and controls.

**Table 1:** Genotype and phenotype used to select cases for recall

Recall by genotype								
Volunteers (n)	Gene	Type	Variants	Effect	Zygosity	Transcript	GnomAD AF	G&H AF
3	*ABCB4*	LoF	S99*	Frameshift	Het	ENSP00000395716.1:p. Ser99Leufs*11	0.00039970	0.00336538
1	*ABCB4*	LoF	F758*	Frameshift	Het	ENSP00000392983.1:p. Leu759Tyrfs*38	.	0.00009610
1	*ABCB4*	LoF	Lys30Glyfs*7	Frameshift	Het	ENSP00000392983.1:p. Lys30Glyfs*7	0.00000408	0.00020243
1	*ABCB11*	LoF	A1044*	Frameshift	Het	ENSP00000497931.1:p. Ala1044Leufs*53	.	0.00009562
1	*ABCB11*	LoF	c.2611-2 A > T	Splice-acceptor variant	Het	ENST00000263817.7:c.2611- 2 A > T	0.00000407	0.00057870
1	*ABCB11*	LoF	W239*	Stop gained	Het	ENSP00000497931.1:p. Trp239*	.	0.00009566
Recall by phenotype (ICP)								
1^1^	*ABCB11*	SNV	R1050H	-	Het	ENSP00000497931.1:p. Arg1050His	0.00000421	0.00019135

^1^Abbreviations: *Het* Heterozygous, *ICP* intrahepatic cholestasis of pregnancy, *LoF* loss of function, *SNV* single nucleotide variant.

**Table 2:** Baseline characteristics and Clinical characteristics of cases and controls

Variable	Cases (*n* = 9)	Controls (*n* = 9)	P value
Demographics			
Age			
*min, max*	34, 57	32, 53	-
BMI (kg/m^2^)	31.31(10.21)	28.70 (6.78)	0.310
Waist-Hip Ratio	0.90 (0.05)	0.90 (0.13)	0.734
Fat percentage (%)	34.90 (12.55)	33.0 (15.85)	0.350
Gender			
*Female*	6 (66.7%)	6 (66.7%)	1.000
Ethnicity			
*British Pakistani*	6 (66.7%)	6 (66.7%)	1.000
*British Bangladeshi*	3 (33.3%)	3 (33.3%)	1.000
Parity			
*Parous*	5 (83.3%)	6(100%)	1.000
Blood tests			
*ALP*	77.0 (41.50)	74.0 (39.50)	0.399
*ALT*	30.0 (24.50)	24.0 (11.0)	0.111
*AST*	22.0 (13.0)	20.0 (10.0)	0.594
*GGT*	26.0 (36.0)	19.0 (8.0)	<0.01*
*Bilirubin*	6.0 (6.0)	6.0 (3.0)	0.964
*AFP*	1.80 (3.05)	1.40 (1.65)	0.097
*LDH*	146 (31.5)	201 (59.0)	0.058
*Bile acids*	0.1 (3.90)	0.1 (3.40)	0.783
*ELF score*	8.57 (1.09)	8.79 (1.67)	0.860
Serum markers of fibrosis			
*FIB-4*	0.80 (0.196)	0.875 (0.20)	0.457
Transient elastography			
*CAP (dB/m) – Mean*	297.00 (67.37)	238.25 (33.66)	0.021*
*LSM (kPA) – Mean*	6.68 (4.25)	4.28 (1.11)	0.072
*Evidence of Severe Fibrosis (F3)*	3/9	0/8 #	0.124
*Evidence of Steatosis (>275 dB/m)*	6/9	2/8 #	0.109

Abbreviations: *AFP* Alpha-fetoprotein, *ALP* Alkaline phosphatase, *ALT* Alanine transaminase, *AST* Aspartate transaminase, *BMI* Body mass index, *CAP* Controlled attenuation parameter, *ELF score* Enhanced liver fibrosis score, *FIB-4* Fibrosis-4 score, *GGT* Gamma-glutamyl transferase, *LDH* Lactate dehydrogenase, *LSM* Liver stiffness measurement. Age, BMI, Waist-Hip Ratio, Fat percentage are presented as Median (IQR, Interquartile range) and p-value obtained using Mann Whitney test. Gender, ethnicity, parity are presented as number (%) and p value was obtained using Fisher’s exact test. Investigations are presented as Median (IQR) or Mean (SD). P value obtained either using t test, Mann Whitney test or Fischer’s exact test. *A p-value <0.05 was considered statically significant. # - 1 control was unable to attend the liver scan.

### Existing Diagnoses

Table 2 describes the clinical and biochemical findings in cases and controls. Out of the 9 cases, 4/9 (44.4%) had a known diagnosis of metabolic dysfunction associated steatotic liver disease (MASLD) of whom 3/4 (75%) had an *ABCB4* LoF and 1/4 (25%) had an *ABCB11* LoF variant (Table 3). 2/9 (22.2%) had a known diagnosis of gallstones both of whom had an *ABCB11* variant (Table 3). No participant with *ABCB4* LoF variants was found to have gallstones.

**Table 3:** Clinical and biochemical findings in cases recalled by genotype and phenotype

	LoF	Demographics	Background	ALT	ALP	GGT	BA	R Factor	ELF score	TE - LSM(kPa) (IQR/med)	TE - CAP (dB/m)	Cholestatic Symptoms (Y/N)	Evidence of sub-clinical cholestatic disease (Y/N)
*ABCB4*	S99*	36–40 F G3P1 BB BMI 31.5	MASLD Pre-diabetes IDA	33	54	26	6	N/A	8.5	4.6 (22%)	357 (17)	Y – RUQ pain	N
	S99*	46–50 F G0P0 BP BMI 36.1	MASLD PCOS Hypercholesterolaemia Fibroids Primary infertility	33	74	27	<2	N/A	8.44	11.6 (38%)	292 (34)	N	N
	S99*	36–40 F G3P2 BP BMI 39.4	No past medical history	20	73	30	<2	N/A	8.47	4.8 (6%)	253 (32)	N	N
	Lys30Glyfs*7	51–55 M BP BMI 31.3	T2DM HTN	60	107	91	8	1.7	9.67	10.2 (25%)	372 (34)	Y – Itching, Dark Urine, RUQ pain	Y – Abnormal LFTs, Abnormal TE
	F758*	31–35 M BP BMI 29.6	MASLD Renal calculus	210	159	267	2	3.9	9.45	4.3 (16%)	334 (48)	Y - Epigastric pain	Y –Abnormal LFTs
*ABCB11*	a1044*	56–60 F G9P7 BB BMI 31.0	MASLD Gallstones Cholecystectomy HTN Hypercholesterolaemia T2DM	30	82	24	<2	N/A	9.64	4.8 (23*%)*	188	Y - Itching in all pregnancies (x7), Steatorrhea, RUQ and epigastric pain	Y – Cholestatic symptoms
	c.2611-2 A > T	41–45 M BP BMI 23.3	HTN Hypercholesterolaemia Renal calculus	24	119	25	<2	N/A	8.57	3.0 (17%)	220	Y – RUQ, and Epigastric pain Steatorrhea	Y – Cholestatic symptoms
	W239*	46–50 F G4P4 BB BMI 42.1	HTN T2DM	17	70	26	2	N/A	8.95	14.3 (26%)	373	Y - Itching during 2nd pregnancy, RUQ/ and epigastric pain	Y – Abnormal TE, Cholestatic symptoms
ICP PHENOTYPE	*ABCB11* SNV R1050H	36–40 F G2P2 BP BMI 25.5	Gallstones ICP	30	77	21	<2	N/A		2.4 (8%)	284	Y – Acute episode of jaundice, itching during pregnancy (ICP)	Y – Cholestatic symptoms, ICP diagnosis

Abbreviations: *ALT* Alanine transaminase (normal range – 10–50 IU/L), *ALP* Alkaline phosphatase (normal range – 40–129 IU/L), *BA* Bile acids (normal range - <10 umol/l, in pregnancy <19–20 umol/l non-fasting), *BB* British Bangladeshi, *BMI* Body mass index, *BP* British Pakistani, *CAP* Controlled attenuation parameter, *ELF score* Enhanced liver fibrosis score, *F/H* Family history, *F* Female, *FIB-4* Fibrosis-4 score, *GGT* Gamma glutamyl transferase (normal range 10–71 IU/L), *HTN* Hypertension, *IDA* Iron deficiency anaemia, *LFT* liver function test, *LoF* Loss of function, *M* Male, *MASLD* Metabolic dysfunctionassociated steatotic liver disease, *PCOS* Polycystic ovary syndrome, *PET* Preeclampsia, *RUQ* right upper quadrant, *SNV* single nucleotide variant, *T2DM* Type 2 diabetes mellitus, *TE* Transient elastography.

### Blood Tests

**Fig. 3:**
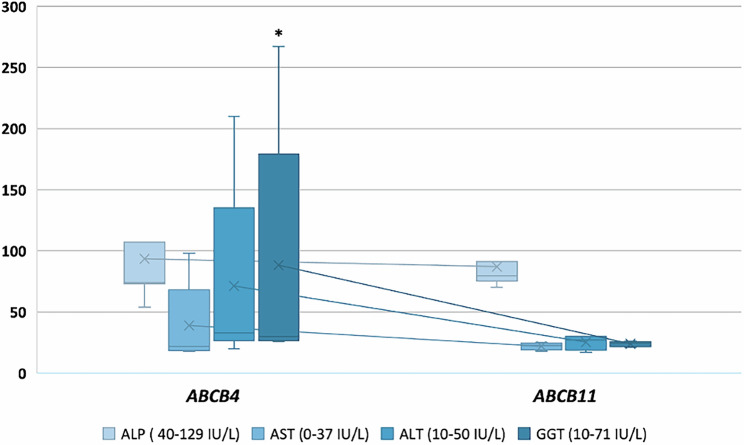
Box Whisker plot illustrating liver blood tests in participants with *ABCB4* and *ABCB11 variants.* Description- Light blue boxes represent ALP (reference range: 40–129 IU/L), medium blue boxes represent AST (reference range: 0–37 IU/L), teal boxes represent ALT (reference range: 10–50 IU/L), and dark blue boxes represent GGT (reference range: 10–71 IU/L). Horizontal black lines inside each box represent the median values, while box boundaries denote the interquartile range (IQR, 25–75th percentiles). Whiskers extend to the minimum and maximum values. Blue crosses (×) represent the mean. An asterisk (*) indicates a statistically significant difference compared with the reference range (*p* < 0.05). Abbreviations: ALP Alkaline phosphatase, AST Aspartate aminotransferase, ALT Alanine transaminase, GGT Gamma-glutamyl transferase, IU/L International units per liter. *n* = 9 biologically independent samples.

Overall, a statistically significant difference was found between cases and controls in the level of GGT. No statistically significant difference was found in other biochemical markers of cholestatic liver disease or clinical fibrosis scores (Table 2). Figure 3 illustrates the distribution of ALP, AST, ALT, and GGT between participants with *ABCB4* and variants *ABCB11* (supplementary data 1). 2/9 (22%) cases had elevated liver transaminases compared to none of the controls. One of the two cases with raised concentrations of liver transaminases, also had elevated ALP levels (Table 3). Both cases with high liver transaminase concentrations had an *ABCB4* LoF variant. No cases with *ABCB11* LoF variants had abnormal liver blood tests.

### Cholestatic Symptoms

Overall, 7/9 (77.7%) cases either reported active or had a previous history of cholestatic symptoms (Table 3). There were six cases who experienced cholestatic symptoms at the time of the recall, 3/6 (50%) cases experienced one cholestatic symptom and 3/6 (50%) experienced two or more cholestatic symptoms. The most common cholestatic symptom reported was RUQ pain or epigastric pain (6/6) followed by steatorrhea (2/6), itching (1/6), and dark urine (1/6) (Table 3).

Of the five parous females, 1/5 (20%) had a confirmed diagnosis of ICP (recalled based on phenotype and the presence of an *ABCB11* SNV R1050H variant). In her first pregnancy, she experienced severe itching with bile acid concentrations exceeding 400 µmol/L, necessitating treatment with UDCA. She underwent an emergency caesarean section at 33 weeks, and the neonate required a 21-day stay in the neonatal intensive care unit (NICU). In her second pregnancy, she was started early on UDCA, with bile acid concentration reaching about 400 µmol/L. This time, she delivered via elective caesarean section at 36 weeks with no NICU admission. Additionally, this participant had a jaundice episode after starting a progesterone only pill. Further, 2/5 (40%) participants had a history suggestive of ICP, reporting significant itching during pregnancy despite no formal diagnoses. In contrast, none of the participants in the control group had cholestatic symptoms during pregnancy or at the time of recall.

### Evidence of Liver Disease

In terms of assessment of hepatic fibrosis through TE, 6/9 cases had an LSM < 8 kPa ruling out fibrosis and 3/9 (33.3%) cases had an LSM > 8 kPa (11.6 (38%), 10.2 (25%), 14.3 (26%))(Table 3). The participant with LSM 11.6 (38%) was excluded from the analysis of the results as IQR/med was >30% (Table 3). All controls had an LSM < 8 kPa. 2/2 (100%) of cases with hepatic fibrosis had an *ABCB11* LoF variant (Table 3).

Hepatic steatosis was identified in 6/9 (66.7%) cases and 2/8 (25%) controls. 4/6 (66.6%) of cases with hepatic steatosis had an *ABCB4* LoF and 2/6 (33.3%) had an *ABCB11* LoF or SNV (Table 3). A statistically significant difference between cases and controls was found in the mean CAP (*p* values = 0.021), indicating a higher degree of steatosis in cases (Table 2).

**Fig. 4:**
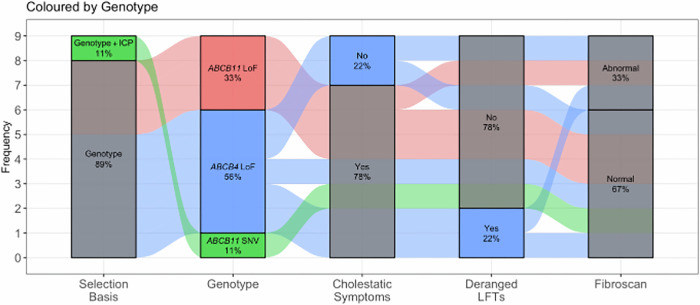
Outcomes of participants stratified by genotype or phenotype recall. Description - Sankey diagram showing the relationship between participant selection, genotype, cholestatic symptoms, LFTs results, and Fibroscan outcomes. Coloured flows represent genotypes: green for ABCB11 SNV or genotype plus intrahepatic cholestasis of pregnancy (ICP), red for ABCB11 LoF variants, and blue for ABCB4 LoF variants. Vertical grey bars represent categorical outcomes at each stage: selection basis, genotype distribution, presence or absence of cholestatic symptoms, deranged or normal LFTs, and Fibroscan status (normal vs. abnormal). Numbers within the boxes indicate the percentage of participants within each category. Flow width is proportional to the frequency of participants in each pathway. Abbreviations: ICP intrahepatic cholestasis of pregnancy, LoF loss of function, LFTs liver function tests, SNV single nucleotide variant. *n* = 9 biologically independent samples.

Overall, 5/9 (55.6%) cases exhibited findings suggestive of liver involvement. Among these, 2/5 (40%) were identified based on abnormal liver stiffness measurements (LSM) on transient elastography (Tables 2 and 3). These individuals had LSM values between 10–4.3 kPa indicating probable or confirmed compensated advanced chronic liver (cACLD).The remaining 2/5(40%) were identified due to currently experiencing two or more cholestatic symptoms (Tables 3) and 1/5 (20%) was classified due to abnormal liver blood tests (Table 3). No control was found to have evidence of cholestatic liver disease. Figure 4 illustrates amongst the cases the relationship between genotype, cholestatic symptoms, blood tests, and fibroscan results.

## Discussion

To the best of our knowledge, in this study we report the results of the first gene candidate recall by genotype and phenotype study associated with cholestatic liver disease in a unique cohort of British Bangladeshi and British Pakistani subjects. Owing to the unique structure of Genes & Health, we were able to recall participants with rare genetic variants suspected to be at high risk of cholestatic liver disease and assess their phenotype. We were able to contribute to the limited evidence base that exists on how heterozygous variants in *ABCB4* and *ABCB11* manifest phenotypically ^11, 15, 36^. We hypothesised that high risk genetic variants predispose to cholestatic liver disease.

We were able to demonstrate that over half of cases (55.6%) had new evidence of liver involvement either due to evidence of fibrosis on TE scan, abnormal liver tests or cholestatic symptoms. With the decreasing cost of genotyping, there arises a promising opportunity to conduct targeted genotyping for individuals at elevated risk of future cholestatic liver diseases, such as patients with ICP and their relatives. We are optimistic that our findings will serve as a catalyst for further genotype-based recall studies. Such studies aim to identify asymptomatic individuals showing signs of cholestatic disease early, enabling timely interventions that may prevent disease progression.

UDCA has been reported in literature that has been used as a treatment in both *ABCB4* and *ABCB11 v*ariants with varying efficiency. For people with variants in hepatobiliary transporters, e.g., *ABCB4*, detected following an ICP diagnosis Hagenbeck et al. recommend lifelong use of UDCA, annual ultrasound studies and monitoring laboratory parameters with the aim of preventing long term sequalae^20, 37^.In addition, in terms of *ABCB4* variants, it has been reported that success by UDCA treatment can be achieved predominantly in the patients with milder phenotypes of the *ABCB4* disease, in which some residual transport capacity is still preserved^14^. In patients with PFIC, prophylactic administration of UDCA may decelerate fibrosis progression and reduce the risk of subsequent complications in some affected individuals^28^. With the growth of various biobanks, there is an emerging opportunity to implement prophylactic UDCA treatment for individuals identified as high-risk due to *ABCB4* or *ABCB11* variants. This proactive approach could potentially mitigate the development of cholestatic liver disease.

There have also been reports of in vitro studies using targeted pharmacotherapy with ivacaftor (initially used in the treatment of cystic fibrosis) for rescuing the function of specific missense variants of the *ABCB4* (G535D, G536R, S1076C, S1176L, and G1178S)^38^ and *ABCB11* (p.A257V, A257V, T463I, and G562D)^39, 40^ which are implicated in various liver diseases. In the context of ICP, Mareux et al. have shown that ivacaftor has therapeutic potential for selected patients with ICP caused by *ABCB11* missense variations and therefore this could potentially be a treatment used in the future in women with ICP as it has been reported that 5% of women with ICP may have a heterozygous *ABCB11* variation^16, 40^.

The application of TE in the general population without known liver disease remains limited, although its utility in a tertiary setting is well-established. According to EASL, non-invasive tests like the FIB-4 index are recommended initially for patients at risk of chronic liver disease in primary care settings. Those with a FIB-4 score of ≥1.30 should undergo liver stiffness measurement using TE. In our study, despite no participant having a FIB-4 score above 1.30, we identified three cases with abnormal liver stiffness measurements. As per EASL guidelines genetic testing is recommended after exclusion of more frequent causes of cholestatic liver disease in adults^41^.

4/9 (44.4%) of the individuals we recalled were previously diagnosed with MASLD. From these four individuals, the presence of hepatic steatosis was confirmed in 3/4 (75%) by performing measuring CAP using TE. One out of the four participants who had an existing diagnosis of MASLD had a normal CAP on TE (Table 3). Interestingly, Nayagam et al. who identified four patients with *ABCB4* variants and MASLD, observed that the two patients who underwent a biopsy exhibited biliary disease without fatty liver disease histologically. Consequently, the original MASLD diagnosis was reconsidered. This finding prompts us to question whether our participants had an accurate diagnosis of MASLD, especially since none underwent a liver biopsy to confirm their conditions.

Hepatic steatosis was identified in 6/9 (66.7%) cases of which 2/6 (33.3%) were found to have LSM > 8 kPa which can indicate hepatic fibrosis. Recent evidence indicates that 12–40% of individuals with MASLD progress to Metabolic dysfunction-associated steatohepatitis (MASH) over a period of 8–13 years, while approximately 15% of patients with MASH and early fibrosis progress to cirrhosis and/or hepatic decompensation within the same timeframe ^42, 43^. In addition, although MASLD was not thought to result in development of progressive fibrosis, fibrosis progression was recently shown to occur not only in MASH, but also in MASLD^44^. Consequently, Pais et al. suggest that practices of no monitoring in patients with steatosis alone might not be appropriate, and a more active, hepatological follow-up could be necessary^44^. In our cohort, it was suggested as per LSM that 2/6 (33.3%) of cases with hepatic steatosis had possible evidence of fibrosis of which 2/2 (100%) participants were not diagnosed with any liver disease at the point of recall. This highlights that if these individuals who were unaware of liver disease were picked up earlier the fibrosis could have been prevented or delayed by lifestyle modifications, such as weight loss as both participants had a BMI > 30.

Although in the literature there is a reported association between *ABCB4* variants and LPAC, none of our cases with *ABCB4* LoF were found to have gallstones ^13, 45^. Whereas previous studies reported no association between *ABCB11* pathogenic variants and gallstones^21^, we report two cases of *ABCB11* (LoF a 1044x, SNV R1050H) with gallstones. Our findings add to the existing body of research on *ABCB11* variants and gallstones, as previously documented in seven Dutch patients. These patients, who presented with gallstones and Benign Recurrent Intrahepatic Cholestasis (BRIC) Type 2, carried missense and splice site variants of the gene^46^. In addition Strubbe et al. reported that in up to one third of patients with BRIC 2 gallstones are observed^47^. BRIC Type 1 and Type 2 are important differential diagnosis we should should consider with our participants with *ABCB11* LoF (a1044x, c.2611-2 A > T, W239x) and *ABCB11* SNV R1050H, especially since BRIC Type 2 has shown to have a correlation with gallstones.

The sole participant formally diagnosed with ICP was recalled due to their phenotype and an *ABCB11* SNV (R1050H). Despite experiencing two severe episodes of ICP, an episode of jaundice after taking a progesterone-only pill, and having gallstones, there was no evidence of cholestatic liver disease present at the time of recall. Monrose et al. reported that the median time from an ICP diagnosis to the onset of liver disease is approximately 13.1 years, underscoring the importance of dedicated long-term follow-up for these patients^48^. Although the participant in our study currently showed no signs of liver disease, they remain at elevated risk for developing cholestatic liver disease in the future. In our cohort, while no other parous females were formally diagnosed with ICP, two of the five parous females—both harbouring *ABCB11* LoF variants—exhibited symptoms suggestive of ICP. Interestingly, none of the parous females with *ABCB4* LoF mutations presented with similar symptoms. This observation contrasts with existing literature that suggests *ABCB4* variants have a greater overall genetic influence on ICP susceptibility than *ABCB11* variants^10^. However, it is important to note that most studies to date have focused on populations of European descent. Consequently, our findings may reflect ancestry-specific variations in disease aetiology, highlighting the need for further research in diverse populations.

Our results have shown discordance between the non-invasive liver blood tests, such as ELF test and FIB-4 and TE as both participants with evidence of fibrosis as shown by TE LSM measurements had normal ELF and FIB 4 (Table 3). As shown by De Silva et al., blood test-based non-invasive liver tests are less accurate in patients of South Asian compared with white ethnicity^49^. However, TE was found to be equally and highly accurate in patients South Asians origin compared with patients of white ethnicity^49^. Therefore, ethnicity must be taken into consideration when assessing for fibrosis.

In addition, we observed that two participants with *ABCB4* variants had abnormal liver blood tests, including elevated levels of ALT, AST, GGT and ALP. In contrast, all participants carrying *ABCB11* variants displayed cholestatic symptoms with no evidence of abnormal liver blood tests. Additionally, a large-scale whole-genome sequencing of the Icelandic population showed an association between *ABCB4* rare variants and increased levels of AST, ALT and GGT, further supporting our observations (24). Abnormal liver blood tests in our study can be potentially explained by *ABCB4* haploinsufficiency, which impedes the neutralisation of bile salts due to reduced biliary phospholipids, thereby damaging the canalicular membrane ^25, 50^. This is consistent with what has been reported in literature that *ABCB4* variants are characterised by higher levels of GGT compared to *ABCB11*^25^. However, as illustrated by other studies, GGT levels alone may not reliably differentiate between cholestasis linked to *ABCB4* and *ABCB11* variants, since some *ABCB4* variants may not show elevated GGT levels^25^. Our observations align with recent research indicating that heterozygous *ABCB4* variants are frequently seen in adults with cholestasis. Notably, a study in Switzerland found that *ABCB4* variants were present in 50% of individuals assessed for unexplained biochemical cholestasis, ICP, or other cholestatic phenotypes. These findings underline the prevalent role of *ABCB4* in various forms of cholestasis and highlight the importance of considering genetic backgrounds when diagnosing and managing these conditions^36^.

This is a small study focussing on genes known to play a role in the aetiology of cholestatic liver disease^11^. We adopted a pragmatic approach to recall *ABCB4* and *ABCB11* LoF variants, given their established impact on protein function. While this approach was tailored to our study’s objectives, it may have introduced some selection bias. We were only able to recall one participant with previous history of ICP. As reported in other recall by genotype (RbG) studies, the small sample size of participants we recalled can lead to a higher variance in results compared with a larger cohort^51^. However, we were able to achieve a 41% recall in a disadvantaged and high risk group which makes this study stand out. Compared to other recall studies, we included a matched control group to allow a direct comparison and reduce the risk of variance. To standardise our approach, we asked participants to be fasted. However, this can affect bile acids as mean, non-fasting serum bile acid concentrations are higher than in fasted individuals^52^.

In addition, most non-invasive tests, such as serum markers of hepatic fibrosis and transient elastography were developed and validated in secondary or tertiary settings and have not been tested for use in primary care or the general population^32^. Despite its utility as a non-invasive tool for assessing liver fibrosis and steatosis, TE has several limitations. TE is operator dependent and requires training and experience for validated quality criteria^53^. Its accuracy can be affected by patient-related factors as LSM can affected by inflammation, venous pressure, cholestasis, amyloid deposition, and food intake and CAP can affected by the BMI^54^. In addition, when obtaining LSM values the reliability needs to evaluated with IQR/Med^54^.

## Conclusion

In summary, we report the first recall-by genotype and phenotype study of individuals identified to be at high risk of cholestatic liver disease based on their genotype or phenotype. Notably, we demonstrated how by first identifying individuals to be at high risk of cholestatic disease by genotype, exploring their phenotype and performing investigations, such as liver blood tests and TE scans we were able to demonstrate evidence of liver involvement and arrange appropriate investigations and follow up. As a result, we hope that integration of genetic information can potentially facilitate personalised medicine according to genotype to make the best therapeutic choice for each individual.

## Supplementary information

Supplementary Information

Description of Additional Supplementary Files

Supplementary Data

Reporting summary

## Data Availability

Genes & Health: Deposition of individual-level whole exome sequencing data from the Genes & Health study is not possible due to the governance model of the cohort and the commitments made to participants. All individual-level data are held securely within the Genes & Health Trusted Research Environment (TRE), and direct export or public deposition of genomic data is not permitted to protect participant confidentiality. Instead, access is available to registered researchers worldwide via application to the Genes & Health Executive (https://www.genesandhealth.org/). Applications are reviewed on a rolling monthly basis, and approved researchers are granted access to the individual-level data within the TRE, where analyses can be conducted and can request the data files used in this study from the corresponding author. GWAS data precomputed for all available phenotypes is freely downloadable under a CC BY-SA licence. This can be accessed using the Google Cloud SDK (gcloud CLI), following the instructions in the documentation, and retrieving the dataset at: gs://genesandhealth_publicdatasets/. We have provided source data for Fig. 3 as Supplementary Data 1. Figures 1 and 2 are flow diagrams with no source data and Fig. 4 is a summary of Table 3.
