# Supplementary material for: A genes and health recall study of intrahepatic cholestasis of pregnancy and cholestatic liver disease
Source: Commun Med (Lond). 2025 Dec 23;5:531. doi: 10.1038/s43856-025-01228-4 (PMC12738555; doi:10.1038/s43856-025-01228-4)
Supplement: Supplementary file 1 — Supplementary Information [file 43856_2025_1228_MOESM1_ESM.pdf]

Supplementary Material

Supplementary Table 1. Summary of volunteers with significant loss of function variants invited for recall by genotype

Volunteers (n)	Gene	Type	Variants	Effect	Zygosity	Transcript	GnomAD AF	G&H AF
10	*ABCB4*	LoF	S99*	Frameshift	het	ENSP00000395716.1: p. Ser99Leufs*11	0.00039970	0.00336538
1	*ABCB4*	LoF	F758*	Frameshift	het	ENSP00000392983.1: p. Leu759Tyrfs*38	.	0.00009610
1	*ABCB4*	LoF	Lys30Glyfs*7	Frameshift	het	ENSP00000392983.1: p. Lys30Glyfs*7	0.00000408	0.00020243
1	*ABCB4*	LoF	R595*	Stop gained	het	ENSP00000392983.1: p. Arg595*	0.00001627	0.00009593
1	*ABCB11*	LoF	A1044*	Frameshift	het	ENSP00000497931.1: p. Ala1044Leufs*53	.	0.00009562
1	*ABCB11*	LoF	c.2611-2A>T	Splice-acceptor variant	het	ENST00000263817.7:c.2611- 2A>T	0.00000407	0.00057870
1	*ABCB11*	LoF	W239*	Stop gained	het	ENSP00000497931.1: p. Trp239*	.	0.00009566

Abbreviations: AF – Allele frequency, G&H – Genes and Health, GnomAD- Genome aggregation database, Het-Heterozygous, LoF- Loss of function. Inclusion criteria (< 1% MAF).

Supplementary Table 2. Summary of volunteers with variants invited for recall by phenotype

Volunteers (n)	Phenotype recall								
	Phenotype	Gene	Type	Variants (SNV)	Zygosity	Transcript	dbSNP	GnomAD AF	G&H AF
1	ICP	*ABCB4*	SNV	G1254S	het	ENSP00000496956.1:p. Gly1254Ser	rs781315185	0.00003656	0.00028843
1	ICP	*ABCB4*	SNV	P1050S #	het	ENSP00000497931.1:p. Asp1284Asn			0.00019146
1	ICP	*ABCB4*	SNV	A833T #	het	ENSP00000496956.1:p. Ala833Thr			0.00009638
1	ICP	*ABCB4*	SNV	N510S	het	ENSP00000496956.1:p. Asn510Ser	rs375315619	0.00019110	0.00057394
1	ICP	*ABCB4*	LOF	S99*	het	ENSP00000395716.1:p. Ser99Leufs*11		0.00039970	0.00336538
1	ICP	*ABCB11*	SNV	D1284N #	het	ENSP00000497931.1:p. Asp1284Asn	rs766784155	0.00001228	0.00028780
1	ICP	*ABCB11*	SNV	R1050H	het	ENSP00000497931.1:p. Arg1050His	rs72549398	0.00000421	0.00019135
1	ICP	*ABCB11*	SNV	V284A	het	ENSP00000497931.1:p. Val284Ala	rs200739891	0.00026040	0.00009558

Abbreviations: AF – Allele frequency, dbSNP - Single nucleotide polymorphism database, G&H – Genes and Health, GnomAD - Genome aggregation database, Het - Heterozygous, ICP – Intrahepatic cholestasis of pregnancy, MAF- Minor allele frequency, LoF - Loss of function, SNV- Single nucleotide variant. Inclusion criteria (< 1% MAF).

Supplementary Table 3. Personal History Template

CHOLESTASIS: itching? /Jaundice? /Dark urine? /Loose fatty stools?/ Pale stools?/RUQ/epigastric pain?

COVID: Any flu-like symptoms or a positive covid test in the last 6 weeks?

PAST MEDICAL HISTORY: Any liver disorders/Any Hepatitis? /Any gallstones?/ Any Malignancy?

PAST SURGICAL: Any operations?/ Any cholecystectomy?

OBSTETRIC HISTORY:
Any previous pregnancies – If yes -? Number
Outcome (live/stillbirth)/Gestation delivered /Method of delivery/Weight of foetus/
Admission in neonatal ICU? Cause for admission?
Any previous maternal complications – If yes - Any previous ICP?/ Any previous pre-eclampsia?/Any previous GDM?
Any previous foetal complications

MEDICATIONS:
Any contraceptive use/Any herbal remedies/Any vitamins?
Any new medications introduced in the last 3 months? Any problems?

ALLERGIES: (to medicines, foods, animals, environment):

SOCIAL HISTORY
Smoking:
Pan chewing:
Alcohol:
Recreational drugs
Occupation:
Home setup:

FAMILY HISTORY TEMPLATE
Any conditions that run in the family:
Any liver disorders?/ Any gallstones/ cholecystectomy?/Any malignancy?
Any problems with pregnancy that run in the family?
