# Supplementary material for: A genes and health recall study of intrahepatic cholestasis of pregnancy and cholestatic liver disease
Source: Commun Med (Lond). 2025 Dec 23;5:531. doi: 10.1038/s43856-025-01228-4 (PMC12738555; doi:10.1038/s43856-025-01228-4)
Supplement: Supplementary file 4 — Reporting summary [file 43856_2025_1228_MOESM4_ESM.pdf]

nature portfolio

Corresponding author(s): Julia Zöllner (j.zollner@ucl.ac.uk)

Last updated by author(s): 10/09/2025

Reporting Summary

Nature Portfolio wishes to improve the reproducibility of the work that we publish. This form provides structure for consistency and transparency in reporting. For further information on Nature Portfolio policies, see our Editorial Policies and the Editorial Policy Checklist.

Statistics

For all statistical analyses, confirm that the following items are present in the figure legend, table legend, main text, or Methods section.

n/a	Confirmed	
☐	☒	The exact sample size (*n*) for each experimental group/condition, given as a discrete number and unit of measurement
☒	☐	A statement on whether measurements were taken from distinct samples or whether the same sample was measured repeatedly
☐	☒	The statistical test(s) used AND whether they are one- or two-sided<br>*Only common tests should be described solely by name; describe more complex techniques in the Methods section.*
☒	☐	A description of all covariates tested
☐	☒	A description of any assumptions or corrections, such as tests of normality and adjustment for multiple comparisons
☐	☒	A full description of the statistical parameters including central tendency (e.g. means) or other basic estimates (e.g. regression coefficient) AND variation (e.g. standard deviation) or associated estimates of uncertainty (e.g. confidence intervals)
☐	☒	For null hypothesis testing, the test statistic (e.g. *F*, *t*, *r*) with confidence intervals, effect sizes, degrees of freedom and *P* value noted<br>*Give P values as exact values whenever suitable.*
☒	☐	For Bayesian analysis, information on the choice of priors and Markov chain Monte Carlo settings
☒	☐	For hierarchical and complex designs, identification of the appropriate level for tests and full reporting of outcomes
☒	☐	Estimates of effect sizes (e.g. Cohen's *d*, Pearson's *r*), indicating how they were calculated

Our web collection on statistics for biologists contains articles on many of the points above.

Software and code

Policy information about availability of computer code

Data collection	Data were inputted into RedCap.
Data analysis	Statistical analysis was performed using SPSS for Mac Version 28 and 29

For manuscripts utilizing custom algorithms or software that are central to the research but not yet described in published literature, software must be made available to editors and reviewers. We strongly encourage code deposition in a community repository (e.g. GitHub). See the Nature Portfolio guidelines for submitting code & software for further information.

Data

Policy information about availability of data

All manuscripts must include a data availability statement. This statement should provide the following information, where applicable:

- Accession codes, unique identifiers, or web links for publicly available datasets
- A description of any restrictions on data availability
- For clinical datasets or third party data, please ensure that the statement adheres to our policy

Genes & Health: Individual-level participant data are available to researchers and industry partners worldwide via application to and review by the Genes & Health Executive (https://www.genesandhealth.org/); applications are reviewed monthly. Approved researchers have access to individual-level data in the Genes & Health Trusted Research Environment (TRE) and can request the data files used in this study from the corresponding author(s). All data exports from the Genes & Health TRE are reviewed to prevent release of identifiable individual-level data. Summary data may be exported for cross-cohort meta-analysis or replication and for

publication, subject to review. UK Biobank: All individual-level data are available to bona fide researchers from the UK Biobank upon application (https://www.ukbiobank.ac.uk/).).

Research involving human participants, their data, or biological material

Policy information about studies with human participants or human data. See also policy information about sex, gender (identity/presentation), and sexual orientation and race, ethnicity and racism.

Reporting on sex and gender	In our study we have categorised participants based on self reported gender identity.
Reporting on race, ethnicity, or other socially relevant groupings	In our study participants self reported their race and ethnicity.
Population characteristics	Cases consisted of participants with heterozygous loss of function (LoF) variants in ABCB4 and ABCB11 (genotype re-call) or with a previous intrahepatic cholestasis of pregnancy (ICP) diagnosis.
Recruitment	Cases were defined as participants with rare (minor allele frequency <1%) heterozygous loss of function (LoF) variants in ABCB4 and ABCB11 (genotype re-call) or with a previous intrahepatic cholestasis of pregnancy (ICP) diagnosis (ICD10 O26.6). Cases were matched 1:1 to controls. Participants were contacted and invited to attend the study.
Ethics oversight	Genes & Health ethical approval was granted by the South East London National Research Ethics Committee (14/LO/1240) in 2014. This recall study operated with ethical approval from the West of Scotland Research Ethics Service (22/WS/0109).

Note that full information on the approval of the study protocol must also be provided in the manuscript.

Field-specific reporting

Please select the one below that is the best fit for your research. If you are not sure, read the appropriate sections before making your selection.

☒ Life sciences ☐ Behavioural & social sciences ☐ Ecological, evolutionary & environmental sciences

For a reference copy of the document with all sections, see nature.com/documents/nr-reporting-summary-flat.pdf

Life sciences study design

All studies must disclose on these points even when the disclosure is negative.

Sample size	Out of the 22 cases invited for recall, 9/22 attended. All cases were matched 1:1 with controls. In total this study include18 participants (9 cases and 9 controls).
Data exclusions	No data were excluded from the analysis. However, of note is that 1/9 cases with raised LSM on TE and high IQR was excluded as having evidence of fibrosis due to IQR >30%.
Replication	No direct replication of the study was conducted.
Randomization	No randomisation was performed. This was a recall by genotype and phenotype study. All cases were matched with controls.
Blinding	This was a single-blinded study. In accordance with Genes & Health guidelines and ethical approvals participants do not routinely receive information about their genotype prior to the re-call visit and therefore participants were blinded.

Reporting for specific materials, systems and methods

We require information from authors about some types of materials, experimental systems and methods used in many studies. Here, indicate whether each material, system or method listed is relevant to your study. If you are not sure if a list item applies to your research, read the appropriate section before selecting a response.

Materials & experimental systems

n/a	Involved in the study
☒	☐ Antibodies
☒	☐ Eukaryotic cell lines
☒	☐ Palaeontology and archaeology
☒	☐ Animals and other organisms
☐	☒ Clinical data
☒	☐ Dual use research of concern
☒	☐ Plants

Methods

n/a	Involved in the study
☒	☐ ChIP-seq
☒	☐ Flow cytometry
☒	☐ MRI-based neuroimaging

Clinical data

Policy information about clinical studies

All manuscripts should comply with the ICMJE guidelines for publication of clinical research and a completed CONSORT checklist must be included with all submissions.

Clinical trial registration	N/A
Study protocol	*Note where the full trial protocol can be accessed OR if not available, explain why.*
Data collection	Patients were seen in Genes and Health fascilities between April 2023 - August 2023.
Outcomes	Our aim was to identify cases at highest genetic risk of cholestatic liver disease and correlate genetic risk with clinical findings. We hypothesised that heterozygous LoF variants or a previous history of ICP would predispose to cholestatic liver disease and may be detectable in advance of symptomatic disease onset.

Plants

Seed stocks	N/A
Novel plant genotypes	N/A
Authentication	N/A
